# Signaling in Fibrosis: TGF-β, WNT, and YAP/TAZ Converge

**DOI:** 10.3389/fmed.2015.00059

**Published:** 2015-09-03

**Authors:** Bram Piersma, Ruud A. Bank, Miriam Boersema

**Affiliations:** ^1^Matrix Research Group, Department of Pathology and Medical Biology, University Medical Center Groningen, University of Groningen, Groningen, Netherlands

**Keywords:** fibrosis, myofibroblast, TGF-β, WNT, YAP/TAZ, Hippo, signaling

## Abstract

Chronic organ injury leads to fibrosis and eventually organ failure. Fibrosis is characterized by excessive synthesis, remodeling, and contraction of extracellular matrix produced by myofibroblasts. Myofibroblasts are the key cells in the pathophysiology of fibrotic disorders and their differentiation can be triggered by multiple stimuli. To develop anti-fibrotic therapies, it is of paramount importance to understand the molecular basis of the signaling pathways contributing to the activation and maintenance of myofibroblasts. Several signal transduction pathways, such as transforming growth factor (TGF)-β, Wingless/Int (WNT), and more recently yes-associated protein 1 (YAP)/transcriptional coactivator with PDZ-binding motif (TAZ) signaling, have been linked to the pathophysiology of fibrosis. Activation of the TGF-β1-induced SMAD complex results in the upregulation of genes important for myofibroblast function. Similarly, WNT-stabilized β-catenin translocates to the nucleus and initiates transcription of its target genes. YAP and TAZ are two transcriptional co-activators from the Hippo signaling pathway that also rely on nuclear translocation for their functioning. These three signal transduction pathways have little molecular similarity but do share one principle: the cytosolic/nuclear regulation of its transcriptional activators. Past research on these pathways often focused on the isolated cascades without taking other signaling pathways into account. Recent developments show that parts of these pathways converge into an intricate network that governs the activation and maintenance of the myofibroblast phenotype. In this review, we discuss the current understanding on the signal integration between the TGF-β, WNT, and YAP/TAZ pathways in the development of organ fibrosis. Taking a network-wide view on signal transduction will provide a better understanding on the complex and versatile processes that underlie the pathophysiology of fibrotic disorders.

## Introduction

Regardless of the initial trigger, chronic organ injury disturbs the cellular and molecular processes of normal wound healing, resulting in organ fibrosis and eventually organ failure (1). Chronic injury causes prolonged activation of effector cells, such as fibroblasts (2), pericytes (3–5), bone marrow-derived cells (6–8), and possibly cells from epithelial (9) or endothelial origin (10, 11), which differentiate toward myofibroblasts. In normal granulation tissue, myofibroblasts are essential for the deposition, contraction, and remodeling of the extracellular matrix (ECM) and thereby promote wound healing (12). However, aberrant wound healing results in increased proliferation and attenuated apoptosis of myofibroblasts. The well-developed cytoskeletal apparatus of myofibroblasts contains actin and myosin, which are linked to the so-called supermature focal adhesions that connect the cells actin filaments to the ECM (13). This allows myofibroblasts to contract the ECM around them and create contractures that impede organ function (14). The *de novo* expression of smooth muscle α-actin (αSMA), an isoform usually expressed in smooth muscle cells, further enhances their contractile capabilities (15, 16). Moreover, myofibroblasts are notorious producers of ECM components, such as collagens, glycoproteins, and proteoglycans, resulting in the formation of fibrous scar tissue. Cross-linking of collagen in fibrous scar tissue makes it highly resistant to protease degradation and results in irreversible scarring and destruction of the tissue architecture (17). Although a large body of knowledge exists on myofibroblast biology, as of to date, no approved therapies are available that can reverse fibrosis (18, 19). Thus, understanding the molecular mechanisms that govern the differentiation and maintenance of myofibroblasts in fibrotic diseases is of paramount importance.

The differentiation of myofibroblasts is governed by an interplay between different mechanisms. Under increased tissue stiffness and mechanical strain, fibroblasts become activated and show increased β- and γ-actin and αSMA-containing stress fibers, linked to focal adhesions (15). They also start to express the ED-A splice variant of cellular fibronectin – crucial for myofibroblast differentiation – at the plasma membrane (20, 21). Membrane protruding integrin molecules connect the ECM components to the actin fibers, which allows for the conversion of mechanical into biochemical cues that are relayed to the nucleus.

Alternatively, myofibroblast differentiation is driven by biochemical signaling of extracellular growth factors. Many growth factor families have been studied extensively in the context of organ fibrosis, with an emphasis on the transforming growth factor (TGF)-β and Wingless/Int (WNT) signaling pathways as key mediators [reviewed in Ref. (22, 23)]. Their mode of action describes the production of soluble growth factor ligands by a variety of cell types. The growth factors are stored in the ECM, until they are activated and released by mechanical tension or proteolytic cleavage, which enables these ligands to engage their membrane-bound receptors. The receptors relay the biochemical signal inwards, via kinase complexes, to the nucleus. Nuclear transcriptional modulators then act on the chromatin complex in order to change the transcriptional landscape, and thereby promote or repress transcription of target genes. Recently, in fibrosis research the attention shifted toward a relatively new signaling cascade: yes-associated protein 1 (YAP)/transcriptional coactivator with PDZ-binding motif (TAZ) signaling. Interestingly, the three mentioned signal transduction pathways have but little molecular similarity but do share one principle: the cytosolic/nuclear regulation of their transcriptional modulators.

In the past, signaling cascades were often studied in isolation, i.e., a ligand signals through its receptor and mediates the nuclear accumulation of one or several transcription factors to modulate target gene expression. This view changed since recent advances suggest that these cascades are in fact organized into complex signaling networks which, dependent on the cellular and environmental context, govern cell function and fate in fibrotic disorders. This inter-pathway communication allows for increased versatility and fine tuning of cellular responses, which may explain the variety of phenotypes found in fibrotic disorders. The aim of this review is to discuss the current understanding on the signal integration between the TGF-β, WNT, and YAP/TAZ pathways in the development of fibrosis. We will start with a short overview of the three pathways, and extend our discussion with a detailed view on how these pathways connect at multiple levels of signal transduction in the context of myofibroblast function and fibrosis. Finally, we touch upon the challenges and considerations in the design of anti-fibrotic therapies, with focus on the cross-talk between the three signal transduction cascades.

## Canonical TGF-β Signaling

**Figure 1:**
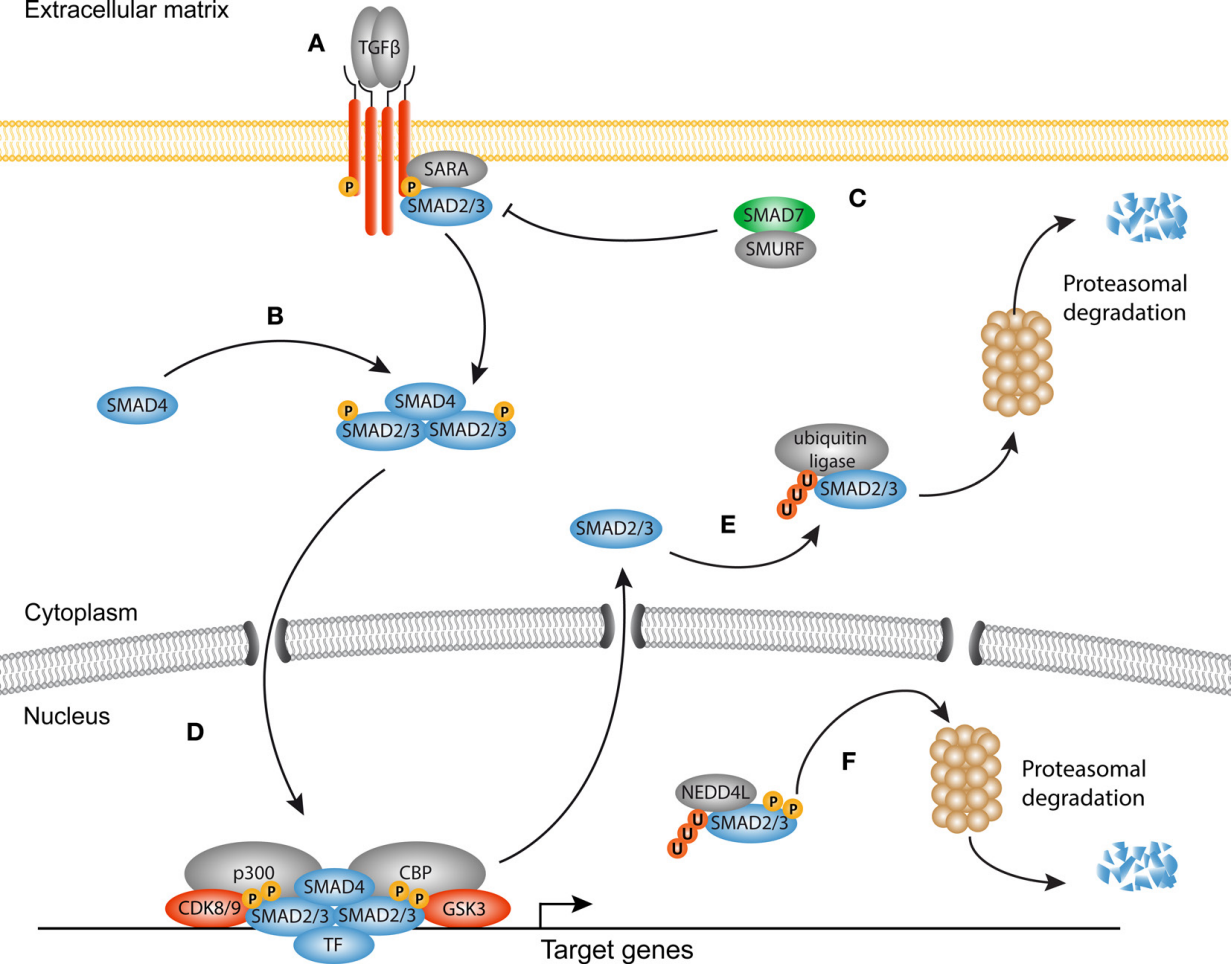
**Canonical TGF-β signaling in fibrosis**. **(A)** Transforming growth factor-β (TGF-β) homodimers engage the type II receptors, which phosphorylate and form a heterotetrameric complex with two type I receptors and additional Smad-binding proteins such as SARA. The signaling domain of the type I receptor mediates phosphorylation and activation of Smad proteins. **(B)** Smad4 associates with phosphorylated Smads to form an active heterotrimeric complex. **(C)** The inhibitory Smad7 together with Smad-specific E3 ubiquitin protein ligases (Smurf) inhibits the receptor complex by targeting it for ubiquitination and proteasomal degradation. **(D)** The activated Smad complex forms a transcriptional module with several transcription factors, co-factors such as p300 and Creb binding protein (CBP) to promote transcription of target genes (e.g., *PAI1, COL1A1, CCN2)*. **(E)** Dephosphorylated Smad proteins continuously shuttle between the nucleus and the cytoplasm. In the cytoplasm, they can be targeted for degradation by ubiquitin ligases. **(F)** Consecutive phosphorylation by CDK8/9 and GSK3 in the nucleus recruits the ubiquitin ligase Nedd4L that target Smad proteins for proteasomal degradation in the cytoplasm, and possibly the nucleus.

The TGF-β superfamily of growth factors consists of multiple proteins that govern a wide range of physiological processes, such as stem cell pluripotency, cell fate determination, proliferation, and differentiation. In humans, over 30 members of the TGF-β superfamily have been documented, including TGF-βs, activins, inhibins, nodal, growth/differentiation factors (GDFs), and bone morphogenetic proteins (BMPs). In this review, we focus mainly on the canonical signaling through TGF-β1, since considerable evidence exists for its role in fibrosis (22) (Figure 1).

In homeostatic conditions, TGF-β is trapped in the ECM together with latency-associated peptides (LAPs) and latent TGF-β-binding proteins (LTBPs) in the so-called large latent complex (LLC) (24). Upon injury, proteolytic cleavage of the LAP (25), or binding of integrins together with increased mechanical forces (26–28), cause release of TGF-β from the LLC allowing it to engage its receptors. Signaling propagation occurs when a TGF-β homodimer interacts with two type I and two type II receptors. Ligand binding initiates the phosphorylation of the SGSGSG domain on the type I receptor by the type II receptor (29, 30). Subsequently, the activated type I receptor is now able to bind and phosphorylate Smad proteins, the central modulators of canonical TGF-β signaling. There are three classes of Smad proteins: regulatory (R)-Smads, co-activator (Co)-Smads, and inhibitory (I)-Smads. R-Smads (Smad2 and Smad3) are phosphorylated by the type I receptor and form heteromeric complexes with the Co-Smad, Smad4. Both R-Smads and Smad4 consist of a N-terminal MH1 and a C-terminal MH2 domain connected by a linker region. Upon phosphorylation of the MH2 domain, Smad complexes shuttle to the nucleus and together with DNA-binding proteins (31–33) localize to specific CAGAC motifs, the so-called Smad-binding elements (SBE), to regulate transcription of target genes (34).

There is ample evidence that TGF-β signaling is a key regulator of myofibroblast biology in the heart, lungs, liver, kidneys, and skin (35–49). TGF-β levels are elevated in fibrotic tissues and myofibroblasts display nuclear accumulation of Smads *in vivo* accompanied with an increased expression of TGF-β target genes and decreased levels of the inhibitory Smad6 and Smad7. Despite a tremendous body of experimental work, the mechanisms underlying Smad-induced fibroblast activation are incompletely understood, as both activation and inhibition of Smads can promote fibrogenesis, dependent on the context (37, 47, 50–55). Furthermore, inhibition of the Smad signaling cascade does not completely attenuate the fibrotic response, which suggests that several other signaling cascades are involved in activating the transcriptional program of myofibroblasts. It has become evident that transcriptional output of Smad signaling is tightly controlled by the interplay with a variety of master transcription factors, DNA-binding (co)factors, repressors, and chromatin readers, and writers (33, 56).

## Canonical WNT Signaling

**Figure 2:**
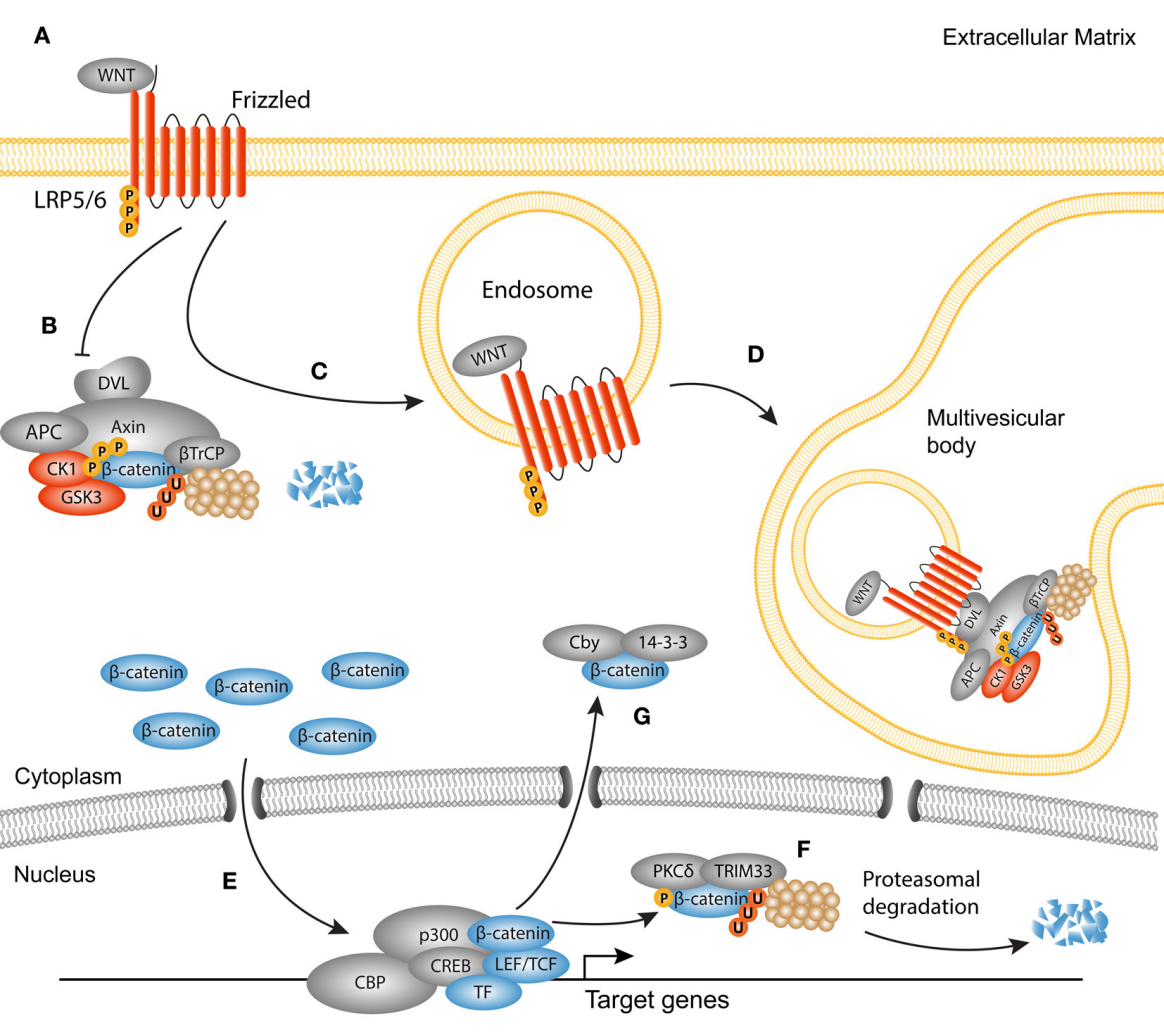
**Active canonical WNT signaling in fibrosis**. Simplified scheme showing the activated canonical WNT cascade and translocation of β-catenin. **(A)** WNT ligands bind to a frizzled receptor and form a complex with the co-receptor Lipoprotein-related-receptor protein (LRP). **(B)** The ubiquitination activity of the destruction complex [comprised of disheveled (DVL) Axin, adenomatous polyposis coli (APC), glycogen synthase kinase 3 (GSK3), and casein kinase 1(CK1)] is inhibited causing β-catenin to accumulate in the cytoplasm. **(C)** As later event, clusters of receptor complexes are internalized into endosomes, which triggers the sequestering of the destruction complex, and subsequent inhibition of GSK3. **(D)** GSK3 is then transported to multivesicular bodies where it cannot interact with cytoplasmic β-catenin, and thus protects β-catenin from proteasomal degradation. **(E)** Newly synthesized β-catenin translocates to the nucleus to interact with LEF/TCF transcription factors and other co-factors, such as p300 and CBP. **(F)** Termination of the WNT/β-catenin signaling cycle may occur through phosphorylation of β-catenin by protein kinase C (PKC)δ and subsequent ubiquitination by tripartite motif (TRIM)33. These steps target β-catenin for proteasomal degradation in the nucleus. **(G)** Another possible route for the termination of β-catenin activation is the cytoplasmic sequestering by 14-3-3ζ and Chibby (Cby).

Discovered in *Sophophora* (Drosophila) as Wingless and in the mouse as Int1, together termed WNT in mammals, canonical WNT signaling comprises the molecular interactions leading to the nuclear translocation of β-catenin [reviewed in Ref. (23, 57)] (Figure 2). WNTs have primarily been studied in fetal development as they are responsible for the formation and polarity of the primary body axis (58), but it has become evident that they are versatile growth factors in both homeostasis and disease. Soluble WNT ligands bind to a family of seven transmembrane receptors named Frizzled (Fz). A single WNT ligand can interact with several Fz receptors, and vice versa (59). In a WNT-off state, the concentration of endogenous WNT antagonists outweighs that of WNT ligands, which results in the phosphorylation of cytoplasmic β-catenin by two subunits from the β-catenin destruction complex, glycogen synthase kinase (GSK)3, and casein kinase (CK)1. These phosphorylation events trigger subsequent ubiquitination and proteasomal degradation of β-catenin (60). In a WNT-on state, ligands engage the Fz receptors which function together with the low-density-lipoprotein-receptor-related proteins (LRP)5 and LRP6 co-receptors to activate the downstream signaling cascade. LRP is phosphorylated in its cytoplasmic tail by GSK3 and CK1 proteins (61–63). The activated Fz/LRP complex interacts with Disheveled (DVL), Axin, and GSK3 through Pro-Pro-Pro-(Ser/Tyr)-Pro repeats (62, 63). Axin functions as a scaffold for the destruction complex, as it directly interacts with β-catenin, GSK3, CK1, the tumor suppressor protein adenomatous polyposis coli (APC), and the ubiquitin ligase β-TrCP. As Axin and GSK3 are sequestered to the plasma membrane by the Fz/LRP complex, β-TrCP is excluded from the destruction complex, limiting β-catenin ubiquitination and degradation (64). An alternative route of WNT activation describes the formation of LRP5/6 aggregates that are internalized together with the destruction complex in the so-called multivesicular bodies. Once inside the multivesicular bodies, a large portion of β-catenin cannot interact with the destruction complex and thus escapes ubiquitination (65, 66). Stabilized β-catenin now accumulates in the nucleus where it associates with T-cell factor/lymphoid enhancer-binding factor-1 (TCF/Lef-1) transcription factors and several co-factors like p300 and CREB binding protein (CBP) to regulate transcription of target genes (67, 68).

About 10 years ago, the first evidence of WNT involvement in myofibroblast biology was found (69). Since then, many studies have emphasized a key role for canonical WNT signaling in fibrogenesis of the heart, lungs, kidneys, and several fibrotic disorders of the skin (70–86). Aberrant activation of WNT signaling can be caused by increased expression of WNT agonists (87), or by silencing of endogenous WNT antagonists, such as proteins from the Dikkopf (DKK) and secreted frizzled-related protein (sFRP) families (77, 86, 88, 89). Experimental models that use exogenous overexpression of WNT ligands or sustained nuclear accumulation of β-catenin suggest that canonical WNT signaling is enough to trigger the expression of a fibrogenic program in fibroblasts (85, 86). However, depletion of β-catenin in the same model could not completely prevent the development of fibrosis, suggesting that β-catenin functions in concert with other pro-fibrotic signals (86). Similar findings come from pulmonary fibroblasts, in which β-catenin stabilization was not sufficient for the upregulation of myofibroblasts-specific genes (74). The discrepancy between the different studies may be due to differences in the constructs used to stabilize β-catenin. Taken together, it has become evident that regulation of β-catenin cytoplasmic/nuclear shuttling is an intricate process, evidenced by the complex expression pattern of WNT ligands in the course of fibrogenesis (71, 77, 82).

## YAP/TAZ Signaling

**Figure 3:**
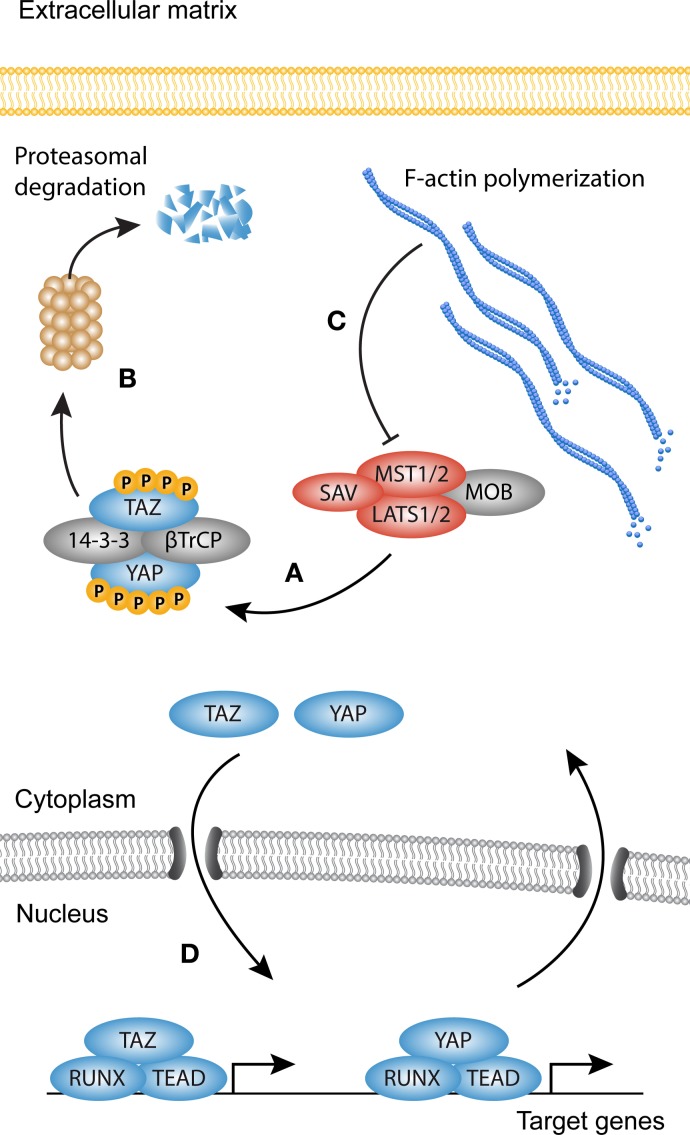
**YAP and TAZ signaling**. Simplified scheme showing activation of YAP and TAZ. **(A)** When the Hippo kinase complex [comprised of Serine/threonine-protein kinases (MST1/2), MOB kinase activator 1 (MOB1), Salvador (SAV), and serine/threonine-protein kinases (LATS1/2)] is active, YAP and TAZ become phosphorylated on multiple sites, creating a so-called phosphodegron. **(B)** Both YAP and TAZ are then sequestered in the cytoplasm by 14-3-3 proteins or targeted for degradation by β-TrCP. **(C)** Polymerization of the F-actin cytoskeleton inhibits the activity of MST1/2, rendering the core kinase complex inactive (several other upstream activators of the core kinase complex are not shown). **(D)** YAP and TAZ now translocate to the nucleus where they associate with transcription factors such as Runt-related transcription factor (RUNX) and TEA domain family member (TEAD) to modulate transcription.

YAP and TAZ are regarded as the main output of the Hippo pathway (Figure 3). YAP and TAZ have been extensively studied in relation to the Hippo core kinase complex, and its role in organ size control, stem cell fate, and cancer (90–92). Both YAP and TAZ contain a WW domain that binds to Pro–Pro–X–Tyr motifs of associated proteins (93–95). The Hippo signaling cascade consists of the Ser/Thr kinases MST1 and MST2, which are orthologs of the Drosophila Hippo kinase (96). MST1/2 binds to Salvador (SAV)/WW45 to form an active enzyme complex that phosphorylates the MOB1A/B subunits of LATS1/2 (97). The activated LATS1/2–MOB1A/B complex in turn phosphorylates YAP and TAZ. The primary phosphorylation of YAP and TAZ triggers subsequent phosphorylation by CK1 kinases. This generates a “phosphodegron” recognized by β-TrCP, leading to YAP and TAZ polyubiquitination and subsequent proteasomal degradation (98). The Serine residues relevant for the inactivation of YAP and TAZ are Ser127 (Ser89 in TAZ) and S381 (S311 in TAZ) (99, 100). When the Hippo pathway is inactive, YAP and TAZ are dephosphorylated and translocate to the nucleus, where they associate with transcription factors and other DNA-binding proteins to modulate target gene transcription. Despite a similar mechanism of activation, YAP and TAZ can bind different transcription factors, but also display overlap as seen with the association with TEA DNA-binding domain (TEAD) transcription factors (101–104). This suggests that their functions only partially overlap, but do share redundancy in some biological contexts.

The capabilities of YAP and TAZ to regulate organ growth and size are striking, but at the same time incompletely understood. It is however clear that they perform these functions, at least in part, as mechanical rheostats independent of the core kinase complex. This becomes evident in cells cultured *in vitro*, which show strong nuclear localization of YAP/TAZ in semi-confluent cultures, but when reaching confluence YAP and TAZ translocate to the cytoplasm (97). A similar biomechanical program can be observed in cells grown on pathologically stiff substrates or substrates that allow cell spreading, as they display predominantly nuclear accumulation of YAP and TAZ and increased transcription of their target genes. By contrast, cells grown on compliant substrates or space limiting substrates display cytoplasmic localization of YAP and TAZ (105–107). F-actin polymerization determines cell morphology and increases in cells cultured in sub-confluence or on stiff substrates. Indeed, F-actin polymerization proves to be the link between cell spreading and YAP and TAZ nuclear translocation (106). The mechanical properties of YAP and TAZ were recently translated to myofibroblast activation and the induction of fibrosis. In biopsies from idiopathic pulmonary fibrosis, both YAP and TAZ levels are elevated, and display a predominantly nuclear localization, which suggests increased transcriptional activity (108). Moreover, YAP and TAZ knockdown in mouse lung and liver fibroblasts cultured on stiff substrates reduces the levels of proteins associated with myofibroblast differentiation such as pro-collagen, αSMA, and plasminogen activator inhibitor (PAI)1 (108, 109). Adding to this, mice heterozygous for TAZ show a remarkable resilience against bleomycin-induced pulmonary fibrosis, possibly due to reduced levels of *CCN2* (CTGF), one of the YAP and TAZ target genes (110). Also in cardiac fibrosis, YAP and TAZ have been a topic of investigation, but whether YAP and TAZ promote fibrogenesis remains elusive, and is probably dependent on the context of injury (111, 112).

Taken together, these findings suggest that the nuclear translocation of YAP and TAZ is the sum of multiple tiers of regulation acting in concert. Because YAP and TAZ activity is not only controlled by Hippo signaling but also by mechanical signals and other signaling cascades, we refer to YAP and TAZ signaling in its broader context.

## Signaling Cascades Converge to Control Fibrotic Processes

In fibrosis, it is becoming clear that the TGF-β, WNT, and YAP and TAZ signaling pathways work in concert, instead of being isolated entities. Several studies have hinted at the inter-pathway cross-talk in the differentiation of myofibroblasts. For instance, in lung fibrosis, protein levels of both YAP and TAZ are increased and have increased nuclear localization (108). This corresponds to the increased levels of nuclear β-catenin and phosphorylated R-Smads found in fibrotic tissues. During skin wound healing in mice, both YAP and TAZ are increased upon injury and translocated to the nucleus. Moreover, TGF-β1 levels are also increased in the dermis, suggesting a link between activation of YAP and TAZ and the production of TGF-β1 (113). Adding to this, YAP- and TAZ-deficient fibroblasts are less reactive to TGF-β stimulation *in vitro*, produced less ECM, have lower expression of myofibroblast markers PAI1 and αSMA, and show lower contractile capabilities (108).

**Figure 4:**
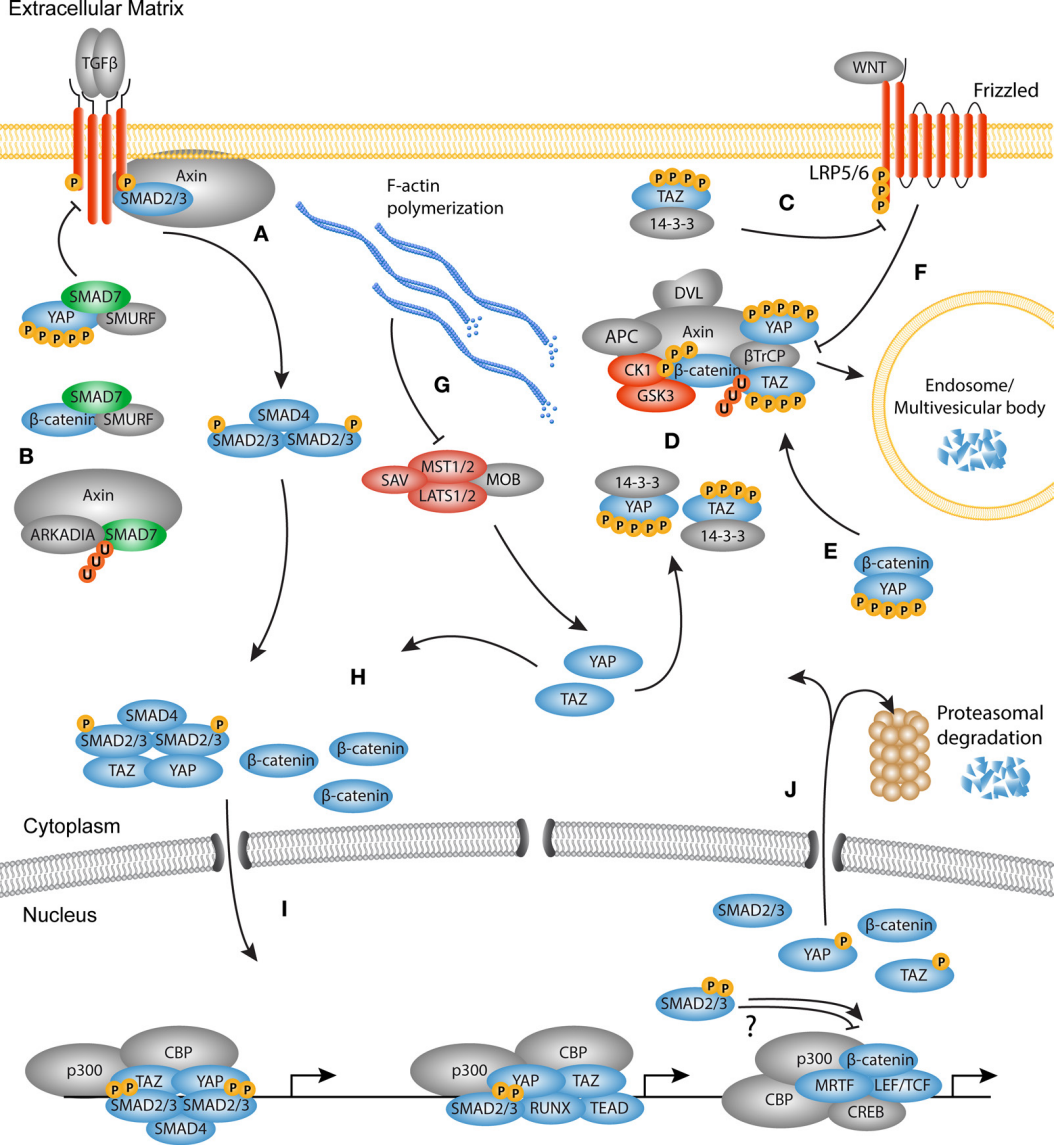
**The TGF-β, WNT, and YAP/TAZ signaling pathways converge**. Schematic overview of the molecular cross-talk between components of the TGFβ, WNT, and YAP/TAZ pathways. **(A)** Upon TGF-β stimulation, Axin promotes the tail-phosphorylation of Smad3. **(B)** Axin also promotes the degradation of inhibitory Smad7, thereby further enhancing the TGF-β signal. Smad7 can associate with both YAP and β-catenin. Binding of YAP to Smad7 increases the affinity for the type I receptor and increases the repressive effects on TGF-β signaling. Smad7 binding with β-catenin can mediate both degradation and stabilization of β-catenin. **(C)** TAZ inhibits the phosphorylation of disheveled (DVL) by casein kinase (CK)1, providing either positive or negative feedback depending on the WNT ligand present. **(D)** The active Hippo core kinase complex phosphorylates both YAP and TAZ creating a phosphodegron. Phosphorylated YAP and TAZ are either sequestered by 14-3-3 proteins or associate with the β-catenin destruction complex. In the destruction complex, YAP and TAZ are necessary for docking of β-TrCP to the complex. **(E)** Phosphorylated YAP also associates with β-catenin to inhibit its nuclear translocation and promote its degradation. **(F)** Upon WNT activation, the destruction complex is inhibited because YAP and TAZ dissociate from the complex. As later event, the destruction complex is sequestered by the LRP/Frizzled/DVL receptor complex and targeted for degradation in the microvascular bodies. **(G)** F-actin polymerization inactivates the core kinase complex, causing YAP and TAZ to be dephosphorylated. Concurrently, β-catenin is not degraded by the inactive destruction complex, so that newly synthesized β-catenin accumulates in the cytoplasm. **(H)** The activated Smad complex associates with YAP or TAZ and translocates to the nucleus. Free and stabilized β-catenin also translocates to the nucleus. **(I)** In the nucleus, the transcription factors may co-localize at the chromatin depending on the context to govern transcription of myofibroblast related genes. **(J)** At the end of the transcription cycle, transcription factors are degraded in the nucleus, or translocate back to the cytoplasm for either degradation or a new round of activation.

The mechanisms through which these pathways communicate are diverse and range from modulating the availability of growth factors and the availability of membrane bound receptors to nuclear entry and activation of transcription factors (Figure 4).

## Pathways Govern Agonist and Antagonist Expression of Other Pathways

The most straightforward form of cross-talk between signaling cascades occurs when activity of one pathway enhances the production of agonists or antagonists of the second. This type of cross-talk can create a feed-forward or feedback loop to enhance or attenuate, respectively, the transcriptional activity of another signaling cascade. For instance, stimulation of fibroblasts with WNT3a enhances the expression of TGF-β1 and subsequent phosphorylation of the MH2 domain of Smad2 (114). Consistently, absence of WNT signaling through LRP5 in bleomycin-induced lung fibrosis decreases the expression of TGF-β1 and attenuates the fibrotic response (115). Reconstitution of active TGF-β1 signaling in LRP5-deficient mice overrides the protective effects of abrogated WNT signaling. Moreover, it was found that TGF-β also enhances WNT signaling through the inhibition of DKK1 (116). Reduced expression of DKK1 enhances the stability and nuclear accumulation of β-catenin in both epithelial cells and fibroblasts, whereas reconstitution of DDK1 *in vivo* attenuates TGF-β-induced fibrosis. This allows cells to communicate over a certain distance and influence the microenvironment of its neighboring cells. Despite the strong effects of altered growth factor signaling, this is often not enough to modulate complex fibrogenic responses. To achieve this kind of complexity, direct interaction between signaling components is required.

## Cytoplasmic Retention and Degradation of Transcriptional Modulators

TGF-β, WNT, and YAP/TAZ output activity all rely on a general mechanism: the nuclear translocation of its transcriptional modulators. To prevent continuous activation, the cell has several means to prevent spontaneous nuclear entry or binding to the DNA. For instance, Smad proteins continuously shuttle between the cytoplasm and the nucleus (33). Without tail-phosphorylation by the TGF-β type I receptor, Smads cannot interact with Smad4 and are unable to engage the DNA. Instead, they are phosphorylated in the linker region by CDK8/9 and GSK3 which renders them susceptible for polyubiquitination and degradation (31). By contrast, β-catenin and YAP and TAZ are sequestered in protein complexes with E-cadherin or 14-3-3 proteins, respectively, or directly targeted for proteasomal degradation by associating with β-TrCP (60, 94, 97).

Recent studies highlighted that extensive cross-talk occurs on the level of cytoplasmic retention and degradation. One example of cross-talk between the YAP and TAZ, and TGF-β pathways is through the interaction with Smad7. As mentioned above, activated Smad proteins need to form a complex with Smad4 in order to become transcriptionally active modules. To regulate the Smad activation cycle, the TGF-β pathway uses Smad7 to form a negative feedback loop via various mechanisms. First, Smad7 can associate with the TGF-β type I receptor. Consequently, R-Smad phosphorylation and complex formation between R-Smads and Smad4 are inhibited (117). Smad7 also recruits E3 ubiquitin ligases, such as Smurf1 and Smurf2, to initiate receptor ubiquitination and degradation of the receptor complex. This self-regulating layer of the TGF-β signaling cascade can be linked to YAP and TAZ signaling, as YAP was found to associate with Smad7 at the type I receptor (118). By binding to YAP, Smad7 has a higher affinity for the type I receptor and increases its repressive effects on TGF-β signaling. Another line of evidence revealed that Smad7 interacts with β-catenin to promote Smurf2-induced mediated ubiquitination and degradation, attenuating WNT activity in the skin (119). By contrast, in cancer epithelial cells it was found that Smad7 promotes the stability of β-catenin by enhancing its association with E-cadherin at the plasma membrane (120). Moreover, upon TGF-β stimulation, the WNT scaffold protein Axin can form a complex with Smad7 and the E3 ubiquitin ligase Arkadia to promote Smad7 degradation (121). These conflicting reports underline that Smad7 may act as repressor or enhancer of cellular signaling depending on the cell type and environmental context.

As mentioned above, the type I receptor initiates phosphorylation of R-Smads, a process that is regulated through the interaction with several adaptor proteins. Interestingly, in unstimulated fibroblasts, Axin facilitates the binding of Smad3 with the type I receptor, independent from the adapter protein SARA (122). Upon TGF-β simulation, Axin promotes the tail-phosphorylation of Smad3 and subsequently dissociates from the type I receptor. Depletion of Axin results in decreased expression of TGF-β responsive genes such as *PAI1*, suggesting that Axin mediates cytoplasmic cross-talk between the TGF-β and WNT pathways which promotes the transcription of pro-fibrotic genes.

Recently it was found that TAZ also communicates with WNT signaling through the interaction of TAZ and β-catenin in the cytoplasm (123). In a WNT-off state, both β-catenin and TAZ associate with β-TrCP and are ubiquitinated and degraded in the proteasome. This process requires active GSK3 phosphorylation of the β-catenin phosphodegron. Upon WNT stimulation, GSK3 dissociates from the destruction complex, β-catenin is dephosphorylated and unable to bind to TAZ. Thus, WNT stimulation induces the stability and nuclear localization of its own transcriptional modulator, β-catenin, as well as TAZ.

Furthermore, inactive YAP and TAZ form a complex together with β-catenin, GSK3, and Axin1 (124). Cytoplasmic YAP/TAZ specifically bind Axin in absence of WNT signals. In this case, Axin facilitates the function of cytoplasmic anchor, as Axin depletion results in a rapid nuclear accumulation of YAP/TAZ. Indeed, stimulation by WNT3a causes dissociation of YAP/TAZ from the destruction complex after which they translocate to the nucleus, and modulate transcription of TEAD target genes. Vice versa, as part of the destruction complex, YAP/TAZ are needed for the docking of β-TrCP to the destruction complex. By releasing YAP/TAZ upon WNT stimulation, β-TrCP cannot bind to the destruction complex and ubiquitinate β-catenin. Furthermore, it was found that protein kinase C zeta (PKCζ) associates with the destruction complex and can phosphorylate YAP and β-catenin on several residues, adding to quick proteasomal degradation (125).

Next to its function in the destruction complex, it was found that TAZ binds to the PY and PDZ domains of DVL2 upon stimulation with WNT3a (126). TAZ binding inhibits the phosphorylation of DVL2 by CK1, which results in reduced β-catenin-mediated activity of LEF/TCF transcription. Interestingly, WNT3a and WNT5a – which in part have opposite functions in β-catenin stabilization – both induce the phosphorylation of CK1, suggesting that TAZ binding to DVL2 may have different outcomes depending on the WNT isoform and receptor pair present (127).

Furthermore, it was found that YAP too fulfills multiple roles in YAP/WNT cross-talk. By directly binding to β-catenin, phosphorylated YAP prevents nuclear translocation of β-catenin and subsequent transcription of LEF/TCF target genes (128). This process is dependent on the activity of the Hippo core kinase complex, as increased Hippo activity induces phosphorylation of YAP and concomitantly reduces levels of β-catenin in the nucleus. Evidence thus shows that the transcriptional modulators of the TGF-β, WNT, and YAP/TAZ pathways are integral factors in the cross regulation between these pathways. Cytoplasmic retention of transcription factors and transcriptional activators proves to be an ingenious system through which the three different pathways tightly regulate their own and each other’s activity.

## Nuclear Shuttling and Transcriptional Modulation

The original view on growth factor signaling described the nuclear accumulation of transcriptional modulators solely as a consequence of ligand-mediated activation. In the absence of a growth factor ligand, Smads and β-catenin were thought to reside exclusively in the cytoplasm and translocate only to the nucleus upon receptor activation. We now know that transcription factor shuttling is not as black and white as once proposed. Without stimulation, R-Smad proteins continuously shuttle between the nucleus and cytoplasm, but display a significant higher concentration in the cytoplasm (129). It is thought that R-Smads reside in the cytoplasm, until TGF-β stimulation releases them for nuclear translocation, enhances their affinity for nuclear importin proteins, and induces nuclear anchoring. In the case of canonical WNT signaling, β-catenin levels are maintained low due to degradation in absence of WNT signals, although β-catenin can be observed in both the cytoplasm and the nucleus. Recent developments reveal that the fold-change in β-catenin levels after WNT stimulation is more important for transcriptional modulation than the absolute levels of β-catenin (130). This finding suggests that even in cells with low basal β-catenin levels, slight changes in nuclear β-catenin are sufficient to initiate transcriptional changes. Nuclear accumulation of YAP and TAZ is governed by the activity of the Hippo signaling cascade as well as biomechanical signals that are relayed from outside the cell. It is becoming clear that fine tuning of nucleocytoplasmic shuttling is not just mediated by a single signaling pathway, but rather by the cross-talk of several components of the TGF-β, WNT, and YAP/TAZ cascades.

In fibrosis, epithelial to mesenchymal transition (EMT) describes the process of epithelial cells that undergo transdifferentiating toward a myofibroblast-like phenotype, a phenomenon observed in both cancer metastasis and fibrosis (9, 131, 132). Upon injury, epithelial cells lose their characteristic cellular junctions and acquire a spindle-like morphology. Cells undergoing EMT often show increased motility, *de novo* expression of αSMA, and elevated expression of ECM components, such as collagens and fibronectin. Also during EMT, several studies have provided evidence that TGF-β, WNT, and YAP/TAZ interact with each other to drive the transformation toward a mesenchymal-like cell type.

One of the first studies, describing the integration of YAP/TAZ and TGF-β signaling in nucleocytoplasmic shuttling, found that TAZ interacts with Smad2/4 and Smad3/4 complexes in epithelial cells (133). The coiled-coil domain in the C-terminal region of TAZ binds to the MH1 domain of Smad2/3 and thereby promotes the nuclear accumulation of Smad2/3 and increases their transcriptional activity on target genes such as *PAI1* and *SMAD7*. Interestingly, low levels of TAZ promote nuclear accumulation, but when the concentration of TAZ increased, it is predominantly located in the cytoplasm and nuclear localization of Smad2/3 is blocked. This suggests that Smad accumulation is strongly dependent on the expression levels and activation status of TAZ.

Similar findings were obtained for YAP, which forms a complex with Smad3, TEAD, and p300 on the *CCN2* promoter in mesothelioma cells (134). Knock down of YAP results in attenuated expression of endothelin1 (*ET1*) and *CCN2*, whereas no immediate differences are seen in the expression of fibronectin and collagens, suggesting that YAP controls the expression of a subset of TGF-β responsive genes. Moreover, levels of metalloproteinase 2 (*MMP2*) are increased upon YAP knock down, strengthening the hypothesis that YAP, together with Smads, governs a pro-fibrotic phenotype. These findings were corroborated in mammary epithelial cells, as YAP/TAZ associates with Smad2/3 and TEADs (135).

A question that then arises is whether transcriptional modulators only need each other for nuclear entry, or also associate with each other at specific promoter or enhancer regions to modulate transcription. Interestingly, during EMT in alveolar epithelial cells, simultaneous stimulation by TGF-β and WNT ligands has synergistic effects on the expression of αSMA as well as the activity of LEF/TCF responsive elements (136). TGF-β alone induces nuclear translocation of β-catenin by inactivating GSK3-mediated degradation, which is further enhanced by WNT stimulation. Delicate ChIP-re-ChIP experiments revealed that β-catenin and Smad3 co-localize at the SBE1 containing region of the αSMA promoter, in a CBP-dependent fashion. These findings are supported by co-localization of β-catenin, Smad3, and CBP in nuclei of epithelial cells in idiopathic pulmonary fibrosis biopsies. Other reports contradict these findings and propose that β-catenin induces the expression of αSMA through interaction with myocardin-related transcription factor (MRTF), a process inhibited by Smad3 (52, 137). One explanation for these conflicting results may be the differences in experimental setup and the different species studied.

Smads and β-catenin were also found to interact on other genes involved in fibrogenesis. As proof of principle, TGF-β and WNT3a synergistically enhanced the promoter activity of sequences containing both SBE and LEF/TCF responsive elements (138). Co-stimulation resulted in a unique expression profile distinct from that seen after stimulation with single growth factors. Interestingly, recent developments describe how YAP can compete with Smad2/3 for promoter occupancy in the transcription of genes involved in mesendoderm differentiation. Gene transcription strongly depends on the phosphorylation status of RNA polymerase II (RNAPII) [reviewed in Ref. (139)]. Briefly, phosphorylation of RNAPII on Ser5 is important for the initiation of transcription, whereas subsequent phosphorylation on Ser2 and Ser7 are crucial for the elongation steps of transcription. β-catenin and LEF-1 associate with enhancer regions of mesendodermal genes such as *MIXL1* and *EOMES* and recruit Ser5 phosphorylated RNAPII to initiate transcription (140). Upon activin stimulation, Smad2/3 localize to the promoter region of these genes to enhance the phosphorylation of Ser2 and Ser7 on RNAPII and thereby promote elongation of transcription. YAP was found to actively inhibit this process by recruitment of the negative elongation factor NELF. Knockdown of YAP reduces the occupancy of NELF and enhances the phosphorylation on Ser2 and Ser7 at target genes, which promotes transcription. Although these results do not directly link to myofibroblast function, they have significant implications on the mechanism by which YAP regulates gene transcription. Future research will reveal if similar mechanisms apply to the regulation of myofibroblast-related genes.

Taken together, TGF-β, WNT, and YAP/TAZ signals converge by modulating the nuclear accumulation and transcriptional activity of their transcription factors. Furthermore, the outcome of this type of cross-talk is not only dependent on the concentration of transcription factors but also on the availability of co-activators and co-repressors, chromatin conformation, and the phosphorylation status of RNAPII, which may vary from one cell type to another (31, 140).

## Transcription Factor Recycling

The final stage of the signal transduction cascades involves the process of transcription factor recycling. In the case of Smad proteins, tail-phosphorylation of the MH2 domain induces nuclear accumulation. Whether nuclear R-Smads engage in transcription or are targeted for nuclear exit and proteasomal degradation relies on a series of phosphorylation and dephosphorylation events by multiple kinases and phosphatases [reviewed in Ref. (141)]. The variety of kinases and phosphatases introduces another level of complexity in the regulation of Smad action, and is greatly dependent on signaling through other pathways at a specific place and time.

One of the examples, through which other pathways interact with Smad recycling describes the temporal regulation by CDK8/9 and GSK3. First CDK8/9 phosphorylate Smad1 on Ser206 and Ser214, which allows binding with YAP and simultaneously triggers phosphorylation by GSK3 on Thr202 and Ser210. The latter phosphorylation events cause YAP to dissociate and Smurf2 to bind with Smad1, and initiate ubiquitination (32). Although Smad1 is not activated by TGF-β but rather by BMPs, one can envision identical mechanisms in the recycling of canonical R-Smads by Nedd4L (31, 142). Whether the association of TAZ with Smad2/3 has similar effects on their recycling remains to be determined. As mentioned above, YAP is able to enhance the repressive functions of Smad7 at the type I receptor. Interestingly, Smad7 has also been found to inhibit TGF-β signaling in the nucleus, where it can use its MH2 domain to bind to DNA sequences containing SBEs (143). DNA bound Smad7 competes with Smad2/Smad4 complexes, thus, directly impairing the transcription of TGF-β responsive genes such as *PAI1*. Whether the interactions between YAP and Smad7 are of importance in this process remain to be elucidated.

## Therapeutic Targeting at the Cross-Roads

Remarkable progress in both biology and pharmacology has led to advances in the development of anti-fibrotic therapies. Many of these therapies aim to target the usual suspects such as ligands and receptors of the TGF-β and WNT signaling cascades using antagonistic antibodies or small-molecule inhibitors. Although the therapeutic efficacy in animal models proves promising (78), trials often fail to achieve significance in clinical endpoints or suffer from severe adverse effects (73) with the exception of one recent study in systemic sclerosis (144). The discrepancy in efficacy between rodents and humans suggests that animal models poorly mimic the pathophysiology of human fibrotic disorders. As we gain insight in the molecular mechanisms that link the TGF-β, WNT, and YAP/TAZ cascades, we come to understand the challenges and pitfalls of targeting one specific signaling pathway [thoroughly reviewed in Ref. (19, 145–147)]. We have seen that signaling cascades are complex and that many pathway components fulfill multiple functions. Because TGF-β, WNT, and YAP/TAZ signals have distinct functions in different cell types and tissues, specific targeting of the fibrotic lesion is crucial. The temporal properties of signal transduction in different phases of disease and homeostasis pose another difficulty in the administration of pathway-wide modulating agents. It is therefore not just a matter of up- or downregulation. For example, targeting of TGF-β or WNT signaling with neutralizing antibodies may have widespread effects on the functioning of several components of the TGF-β, WNT, and YAP and TAZ signaling pathways, as well as numerous other growth factor cascades. To circumvent the wide-spread effects of growth factor inhibition and limit adverse effects, we increasingly depend on the development of small-molecule intracellular inhibitors.

The small-molecule intracellular inhibitor LY2157299 specifically targets the kinase pocket of the type I receptor of the TGF-β cascade without inhibiting the type II receptor and thereby attenuates Smad2/3-dependent transcription of target genes (145). However, LY2157299 has not been studied in clinical trials to halt or reverse fibrosis. Examples of small-molecule intracellular inhibitors for the WNT pathway, such as PKF115–584 and CGP049090 (148), act on the association between β-catenin and LEF/TCF transcriptions factors. Other strategies focus on inhibition of the PDZ domain of DVL or the transcriptional co-activators CBP and p300 (145). By inducing a shift from β-catenin association with p300 to CBP, genes such as *COL1A1* may be negatively regulated (149). However, other studies report that inhibition of β-catenin–CBP also ameliorates fibrosis, suggesting that there is no such thing as pure “good” and “bad” β-catenin signaling (73, 78). As YAP and TAZ have but recently been linked to fibrogenesis, no clinical trials have been performed. Thus, whether targeting of YAP and TAZ is a fruitful strategy against fibrosis progression remains to be elucidated. One of the challenges in the targeting of YAP and TAZ is that they do not possess catalytic domains, but rather depend on specific protein binding domains for the interaction with their binding partners such as LATS1/2, Src family kinases, and TEADs. Nonetheless, a recent study described a potent inhibitor of YAP–TEAD: the benzoporphyrin derivative verteporfin (150). Verteporfin is currently used in the clinic for the treatment of macular degeneration, which makes it appealing for the use in clinical trials for fibrosis. Although inhibition of the YAP–TEAD complex seems a promising anti-fibrotic strategy, as several pro-fibrotic genes are not under control of TEADs, this may prove not to be the best approach.

The disadvantages of pathway-wide molecular inhibitors challenge the scientific community to develop specific targeting strategies against intracellular processes and protein–protein interactions. The increasing insight in the molecular cross-talk between signaling cascades adds new possibilities in drug development. Additionally, by focusing on the elucidation of the crystal structures of protein complexes, we can pursue the rational design of novel small molecular inhibitors to interfere at the cross-roads of signal transduction cascades.

## Conclusion

Recent advancements in the field of TGF-β, WNT, and YAP/TAZ signaling have revealed that these signaling entities do not act alone. The notion that pathway components can have multiple and even opposed functions within one cell partly explains how the inhibition of a single molecular target often does not result in the desired therapeutic effect. This does not only add to the mere understanding of fibrotic processes, but also promotes the necessity to develop highly specific small-molecule intracellular inhibitors that act on protein–protein interactions at the cross-roads of signaling cascades. It should be noted that several of the studies described in this review used artificial ectopic expression of the proteins investigated. This may introduce artifacts that can influence the activity and functionality of the signaling cascades involved. Thus, more detailed studies in representative models for fibrosis focusing on endogenous proteins are required to completely understand the molecular cross-talk *in vivo*. Broadening our view on signal transduction will provide a better understanding of how a limited set of growth factors is able to govern the complex processes that underlie the physiology and pathology of fibrotic disorders.

## Author Contributions

BP, RB, and MB designed the manuscript. BP and MB collected literature and BP drafted the manuscript. All authors critically discussed and revised the content of the manuscript and had final approval of the manuscript in its present form.

## Conflict of Interest Statement

The authors declare that the research was conducted in the absence of any commercial or financial relationships that could be construed as a potential conflict of interest.
